# Supplementary material for: Spatial Variation in Stream-Water Bacterial Communities Across Sites Differing in Wild Chinese Giant Salamander Detection Frequency
Source: Biology (Basel). 2026 Sep 18;15(18):1647. doi: 10.3390/biology15181647 (PMC13604818; doi:10.3390/biology15181647)
Supplement: Supplementary file 1 [file biology-15-01647-s001.zip › Data Related to “Spatial Variation in Stream-Water Bacterial Communities across Sites Differing in Wild Chinese Giant Salamander Detection Frequency”/Microbial Data/result/08.FunctionPrediction/picrust2/heatmap/sample/heatmap.sample.level1.cluster.pdf]

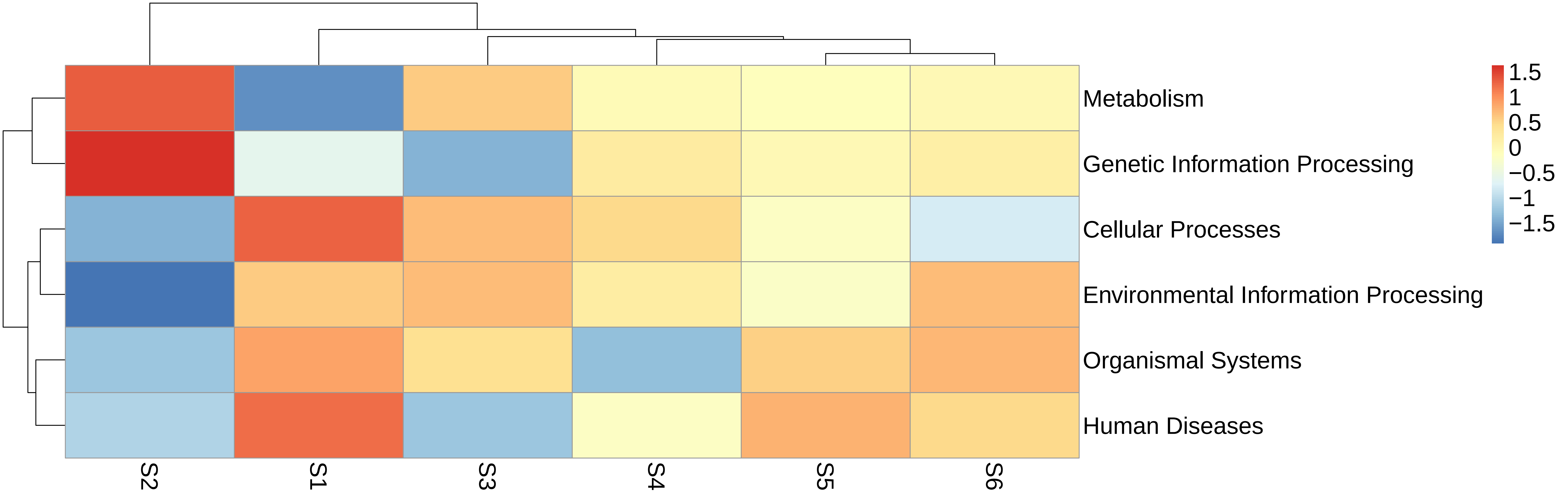
Metabolism
Genetic Information Processing
Cellular Processes
Environmental Information Processing
Organismal Systems
Human Diseases
S2
S1
S3
S4
S5
S6
1.5
1
0.5
0
−0.5
−1
−1.5
